# Enhanced cellular infiltration of human adipose-derived stem cells in allograft menisci using a needle-punch method

**DOI:** 10.1186/s13018-016-0467-x

**Published:** 2016-10-28

**Authors:** Rachel C. Nordberg, Adisri Charoenpanich, Christopher E. Vaughn, Emily H. Griffith, Matthew B. Fisher, Jacqueline H. Cole, Jeffrey T. Spang, Elizabeth G. Loboa

**Affiliations:** 1 Joint Department of Biomedical Engineering, University of North Carolina Chapel Hill and North Carolina State University, 911 Oval Drive, EB III suite 4208, Box 7115, Raleigh, NC 27695 USA; 2 Department of Statistics, North Carolina State University, 2311 Stinson Drive, Box 8203, Raleigh, NC 27695 USA; 3 Department of Orthopaedics, University of North Carolina School of Medicine, 3141 Bioinformatics Building, Chapel Hill, NC 27599 USA; 4 College of Engineering, University of Missouri, W1051 Thomas & Neil Lafferre Hall, Columbia, MO 65211 USA

**Keywords:** Meniscus, Allograft, Human adipose stem cells, Knee reconstruction, Autologous, Stem cells

## Abstract

**Background:**

The meniscus plays a crucial role in knee joint stability, load transmission, and stress distribution. Meniscal tears are the most common reported knee injuries, and the current standard treatment for meniscal deficiency is meniscal allograft transplantation. A major limitation of this approach is that meniscal allografts do not have the capacity to remodel and maintain tissue homeostasis due to a lack of cellular infiltration. The purpose of this study was to provide a new method for enhanced cellular infiltration in meniscal allografts.

**Methods:**

Twenty medial menisci were collected from cadaveric human sources and split into five experimental groups: (1) control native menisci, (2) decellularized menisci, (3) decellularized menisci seeded with human adipose-derived stem cells (hASC), (4) decellularized needle-punched menisci, and (5) decellularized needle-punched menisci seeded with hASC. All experimental allografts were decellularized using a combined method with trypsin EDTA and peracetic acid. Needle punching (1-mm spacing, 28 G microneedle) was utilized to improve porosity of the allograft. Samples were recellularized with hASC at a density of 250 k/g of tissue. After 28 days of in vitro culture, menisci were analyzed for mechanical, biochemical, and histological characteristics.

**Results:**

Menisci maintained structural integrity and material properties (compressive equilibrium and dynamic moduli) throughout preparations. Increased DNA content was observed in the needle-punched menisci but not in the samples without needle punching. Histology confirmed these results, showing enhanced cellular infiltration in needle-punched samples.

**Conclusions:**

The enhanced infiltration achieved in this study could help meniscal allografts better remodel post-surgery. The integration of autologous adipose-derived stem cells could improve long-term efficacy of meniscal transplantation procedures by helping to maintain the meniscus in vivo.

## Background

The meniscus plays a crucial role in knee joint function by providing joint stability and allowing shock absorption, load transmission, and stress distribution within the knee joint. Meniscal tears are the most common knee injuries with an annual reported incidence of 60–70 per 100,000 persons [1, 2]. With limited natural repair capabilities, surgical treatment is very common. Over 1 million surgeries involving the meniscus are performed annually in the USA [3]. Although meniscus repair is preferred, not all meniscus tears can be repaired, such as those that occur in the avascular inner-third and complex tears that compromise the structural integrity of the meniscus. If repair is not possible, a meniscectomy is commonly used to alleviate symptoms. However, partial or total removal of the meniscus has detrimental effects on the knee joint, and these treatments increase the contact stresses on the articular surface of the knee joint [4]. In a knee with a meniscectomy, the contact area between the tibia and the femur is reduced by 50 % [5]. Long-term, there is a high risk of osteoarthritis development after meniscectomy procedures [6].

Currently, the standard treatment for a symptomatic patient who has undergone a substantial meniscectomy is meniscal allograft transplantation [7]. In general, the current body of literature is supportive of meniscal allografts and many patients have experienced reduced pain and improvements in joint function [8]. In addition, meniscal allografts can restore biomechanics of the knee after meniscectomy [9]. Meniscus allograft transplants have shown favorable results in terms of clinical improvement by reducing pain and improving function in both short- and medium-term follow-up, (2 and 5 years, respectively) and even, in some cases, at long-term (>10 years) follow-up [10–13]. The survival rate for a 10-year follow-up in cryopreserved and fresh-frozen meniscal allograft transplants is between 50 and 70 %, with defined failure as tearing and/or sub/total destruction requiring repair/partial meniscectomy or removal of the allograft [10, 13–15]. A magnetic resonance imaging study on the width and thickness of fresh-frozen meniscal transplants showed that shrinkage in the width (89 %) and increase in the thickness (115 %) will be observed within the first year of transplantation [16]. Furthermore, long-term effectiveness in patients who participate in contact sports is unclear; some of the current studies advise against participation in sports with strenuous cutting and twisting [8]. Moving forward, research must address how to better integrate the meniscal allografts into the host knee and prevent degradation over time due to lack of cellular incorporation after implantation [17, 18].

Previous human retrieval studies and animal studies have reported incomplete cellular incorporation, absence of cell proliferation, and a microscopic immune response [19–21]. The high tissue density of the meniscus has been postulated to result in low cellular incorporation in meniscal allografts [22]. Without successful ingrowth and cellular re-population, meniscal allografts lack the capacity to remodel and perform necessary internal maintenance. To improve the success rate of allograft transplants, previous investigators have used animal models to evaluate whether removal of donor cellular components and/or increasing porosity of a transplanted meniscus will allow for the incorporation of a biologically active substrate (growth factors, platelets, or mesenchymal stem cells (MSC)) [23–27]. Importantly, the incorporation of MSC has been suggested to enhance meniscal regeneration and healing [24, 26].

To date, little research [28, 29] has focused on the possibility of modifying the human meniscus with human stem cells to enhance the meniscus as a potential scaffold and/or to determine biological responses of human stem cells seeded on a human meniscal allograft. In this study, human menisci were decellularized to remove donor cells, and meniscus porosity was enhanced via both chemical and mechanical factors. Needle punching was implemented to increase porosity and improve cell penetration. Because of their chondrogenic differentiation potential, autologous availability, and immunocompatibility [30–35], human adipose-derived stem cells (hASC) were utilized to investigate their potential for cell seeding, viability, and migration within the allograft-derived meniscal scaffold. Material property testing was performed to determine the effects of chemical and mechanical decellularization and porosity enhancement on meniscus mechanical properties both pre- and post-hASC recellularization. We hypothesized that increasing the meniscal porosity would improve cellular incorporation of human adipose stem cells without compromising the bulk biochemical or biomechanical integrity of the allograft.

## Methods

### Meniscus acquisition and experimental groupings

Gamma-sterilized, frozen human menisci attached to a hemiplateau were provided by the International Institute for the Advancement of Medicine, a subsidiary of the Musculoskeletal Transplant Foundation (Edison, NJ). Demographic information of menisci used in this study is included in Table 1. The menisci were examined prior to testing and did not exhibit any overt structural defects. Menisci were then removed in their entirety from the hemiplateau prior to laboratory work.

**Table 1:** Demographic information of menisci used for study

Patient #	Age	Gender	Right meniscus	Left meniscus
1	25	Female	Control	–
2	41	Male	–	Control
3	46	Male	Control	–
4	38	Male	–	Control
5	47	Male	Decell + hASC	Decell
6	45	Male	Decell	Decell + hASC
7	39	Male	Decell + hASC	Decell
8	26	Female	Decell	Decell + hASC
9	43	Male	Decell + NP + hASC	Decell + NP
10	38	Male	Decell + NP	Decell + NP + hASC
11	41	Male	Decell + NP + hASC	Decell + NP
12	41	Male	Decell + NP	Decell + NP + hASC

For this study, 20 medial menisci from 12 donors were separated into five experimental groups for an *n* = 4 in each grouping. The groups consisted of (1) control native menisci (control), (2) decellularized menisci (decell), (3) decellularized menisci seeded with hASC (decell + hASC), (4) decellularized needle-punched menisci (decell + NP), and (5) decellularized needle-punched menisci seeded with hASC (decell + NP + hASC). For the control group, only one medial meniscus was used per donor; for the other four groups, both medial menisci were used, split between either the needle-punched or non-needle-punched groups (one for decellularized and one for decellularized + hASC). Following decellularization and needle punching, the four experimental conditions were cultured for 4 weeks after their respective treatments. After 4 weeks of culture, samples were dissected for histological, biochemical, and mechanical analysis.

### Decellularization and porosity enhancement of human meniscus

Chemical decellularization of the menisci was performed to remove donor cells, as described previously [23]. In brief, whole meniscus was placed in deionized water at 37 °C for 48 h, followed by a 24-h enzymatic digestion in 0.05 % trypsin EDTA, a 24-h trypsin neutralization in complete growth medium (CGM) (α-MEM supplemented with 10 % fetal bovine serum, 2 mM l-glutamine, 1 % penicillin-streptomycin), and a 48-h incubation in 2 % Triton X-100 and 1.5 % peracetic acid. The menisci were then subjected to three washes in deionized water. This approach has previously been shown to remove cell debris fully, increase porosity, and maintain mechanical properties and glycosaminoglycan (GAG) content in ovine menisci [23]. To enhance porosity further in the needle-punched groups, a 28 G microneedle was used to punch pores manually through the meniscus in both a superior-to-inferior and inferior-to-superior pattern (1-mm spacing, full thickness punches).

### Cell seeding and cell culture

Human ASC were isolated from liposuction aspirates of female patients undergoing voluntary liposuction procedures using an approved IRB protocol at the University of North Carolina at Chapel Hill (IRB 04-1622), as described previously by our laboratory [36]. CGM was used to expand hASC. An age-grouped (age 24–36, five donors), hASC superlot was generated and verified as representative of individual donors, as described previously [36]. The hASC superlot was seeded at a density of 250,000 cells per gram of meniscal tissue. To enhance cell attachment and integration throughout the meniscus, samples were sealed in sterile heat-sealed bags for seeding and centrifuged five times at 2000 rpm for 1-min intervals, with gentle resuspension of unattached cells after each cycle to increase cell-meniscus contact. After centrifugation, bags were left on an orbital shaker overnight in incubator before the menisci were transferred to specimen cups for remainder of cell culture. Cell-seeded menisci were cultured in an incubator for up to 4 weeks in CGM at 37 °C and 5 % CO_2_. After 4 weeks, cell viability was verified via Calcein AM for visualization (Molecular Probes, Eugene, OR).

### Scanning electron microscopy analysis

To detect changes in ECM after treatments, scanning electron microscopy (SEM; JEOL JSM-6400F, MiQro Innovation, Québec, Canada) was used to visualize specimens before and after decellularization. Menisci were fixed with 2.5 % glutaraldehyde, followed by serial ethanol dehydration. They were then critical point dried in liquid CO_2_ and gold-platinum sputter coated for scanning electron microscopy analyses.

### Histological analyses

**Fig. 1:**
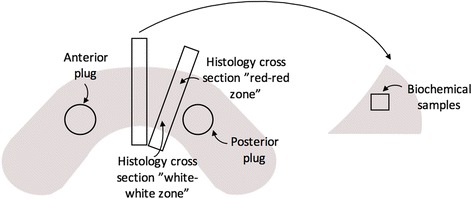
Diagram of meniscus sample preparation. Five-millimeter plugs were taken from the anterior and posterior for material property testing. Biochemical samples were taken beneath the surface of the meniscus. Histological samples were taken in cross sections and imaged in the “red-red zone” towards the exterior of the meniscus and at the “white-white zone” towards the interior of the meniscus

Cross-sectional samples of each meniscus were taken for histological analyses (Fig. 1). To verify the decellularization process, cell-seeded menisci were dissected and fixed in 10 % formalin for 24 h, followed by two washes in phosphate-buffered saline (PBS), ethanol dehydration, and paraffin embedding. Menisci were sectioned into 10-μm thick slices with a Leitz 1512 rotary microtome (Leica, Wetzlar, Germany), and sections were stained with 4,6-diamidino-2-phenylindole (DAPI) to identify residual nuclear debris. Additional sections were stained with fast green/safranin O with a hematoxylin counterstain to visualize collagen organization, proteoglycans, and cellular infiltration in both the “red-red zone” and “white-white zone” of the menisci.

### Biochemical analysis

Menisci were dissected with a scalpel to obtain four samples (approximate size of 1 mm^3^) from beneath the surface of each meniscus (Fig. 1). Samples were lyophilized for 48 h and massed to obtain dry weight of the tissue. Samples were papain digested for 16 h, and DNA content was quantified using a Hoechst 33342 assay (Thermo Fisher, Waltham, MA).

### Mechanical testing

Full-thickness cores were taken from the anterior and posterior regions of the meniscus (Fig. 1), in a direction perpendicular to the femoral surface, using a 5-mm diameter cylindrical biopsy punch. To provide parallel surfaces for even load distribution during testing, the superior and inferior edges of each sample were trimmed using a HM525 NX cryostat (Thermo Fisher, Waltham, MA). The samples were stored at −20 °C until testing and then thawed individually in PBS at room temperature just prior to testing. The initial thickness and diameter were each measured in three locations on the sample using digital calipers, and the mean values were calculated.

Samples were submerged in a PBS bath within a custom fixture, and the static and dynamic mechanical properties were measured in unconfined compression [37] between two rigid, impermeable platens using an axial testing system (Bose EnduraTEC ELF3220, EnduraTEC Systems Corp., Minnetonka, MN) equipped with a 500-g load cell (Model 31 Miniature Load Cell, Sensotec, Columbus, OH). Several compression tests were performed in succession, as follows. A compressive tare load of 0.025 N was applied in load control for 5 min, and then a new sample thickness was calculated based on the initial thickness and the change in actuator displacement under the creep tare load. A stress relaxation test was performed to 10 % strain (based on the post-creep thickness), applied at a rate of 0.01 mm/s and held for 40 min to ensure the sample reached equilibrium. Sinusoidal cyclic loading was then applied using a magnitude of 10 ± 1 % strain (9–11 % strain) for 10 cycles each at frequencies of 0.1, 1, and 10 Hz. A second stress relaxation test was performed to 20 % strain, applied at a rate of 0.01 mm/s and held for 40 min, followed by a second set of sinusoidal cyclic loading to 20 ± 1 % strain (19–21 % strain) for 10 cycles each at frequencies of 0.1, 1, and 10 Hz. Load and displacement were recorded for all tests.

Testing data were processed in MATLAB (MathWorks, Natick, MA). Stress and strain were calculated from the original cross-sectional area and post-creep thickness, respectively. Using the stress relaxation data sets, equilibrium stress was computed for 10 and 20 % strain, defined when the mean stress change per minute was less than 1 %. Compressive equilibrium moduli were determined from the equilibrium stress and applied strain values. Dynamic compressive moduli were measured at each frequency as the mean slope of the stress-strain curves for all cycles plotted together at each strain level.

### Statistics

Statistical analysis was performed following biochemical and mechanical testing. Biochemical and mechanical testing data were analyzed using a linear mixed-effects model and a significance level of 0.05 (SAS 9.4, SAS Institute Inc., Cary, NC). A random blocking factor was introduced to account for correlations between the two menisci taken from the same donor that were divided between treatments. Treatment groups were divided evenly across the blocks to make a complete block design. For biochemical data, the average DNA content of all four samples from within menisci used and the treatment group was treated as a fixed effect. Orthogonal contrasts were used to test: (1) the effect of decellularization on non-needle-punched menisci (control vs. decellularized); (2) the effect of decellularization on needle-punched menisci (control vs. decellularized needle-punched); (3) the effect of reseeding non-needle-punched menisci (decellularized vs. decellularized + hASC); and (4) the effect of reseeding needle-punched menisci (decellularized needle-punched vs. decellularized needle-punched + hASC).

For mechanical data, treatment group was treated as a fixed effect, and anatomical location (anterior, posterior) was treated as a repeated measure. Orthogonal contrasts were used to test: (1) the effect of decellularization (all treatments vs. control); (2) the effect of needle punching (decellularized vs. decellularized needle-punched, decellularized + hASC vs. decellularized needle-punched + hASC); and (3) the effect of seeding with hASC (decellularized vs. decellularized + hASC, decellularized needle-punched vs. decellularized needle-punched + hASC).

## Results

### Decellularization of human menisci

**Fig. 2:**
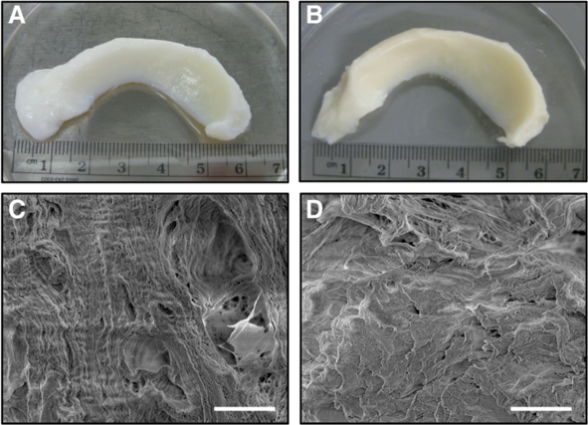
Gross inspection of human meniscus **a** before decellularization and **b** after decellularization showed that overall shapes were maintained for decellularized compared to intact meniscus. Scanning electron micrographs of **c** control meniscus at ×500 magnification, **d** decellularized meniscus at ×500, showing that the extracellular matrix retained its structure throughout the decellularization process (scale bars = 50 μm)

**Fig. 3:**
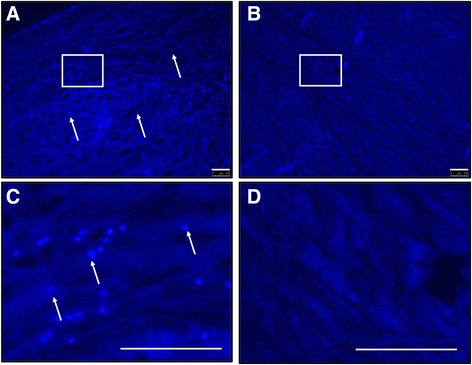
Confirmation of the decellularization process. Cellular and nuclear content was tested via DAPI staining. Bright, punctate spots (*arrows*) indicating nuclei **a** can be seen before decellularization but **b** are absent after decellularization (*blue background* staining of tissue is still present). High-magnification images of nuclear staining in both **c** control and **d** decellularized menisci. (All scale bars = 100 μm)

The general shape and architecture of the menisci were maintained after decellularization (Fig. 2a, b). Electron microscopy revealed that chemical decellularization removed the donor cell membrane but did not unpack the collagen bundle (Fig. 2c, d). Successful removal of donor nucleic acid was observed with negligible DAPI/nuclear staining in the decellularized menisci relative to the intact menisci (Fig. 3).

### Viability of hASC on decellularized menisci

**Fig. 4:**
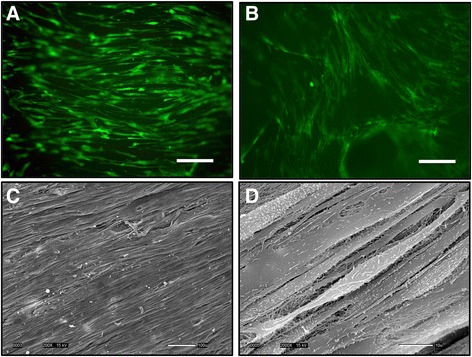
The seeded hASC remained viable and aligned along the fibers of the meniscus. Live staining (*green*) of **a** hASC-seeded decellularized meniscus and **b** needle-punched decellularized meniscus (scale bars = 200 μm). Scanning electron micrographs revealed that hASC were well organized and aligned parallel to the collagen fiber extracellular matrix of the decellularized meniscal allograft, as shown at **c** ×200 (scale bar = 100 μm) and **d** ×2000 (scale bar = 10 μm)

Live staining indicated that hASC remained viable in both decellularized and needle-punched decellularized menisci (Fig. 4a, b). SEM revealed elongated hASC aligned parallel to the native collagen fibers of the decellularized menisci (Fig. 4c, d).

### Histology

**Fig. 5:**
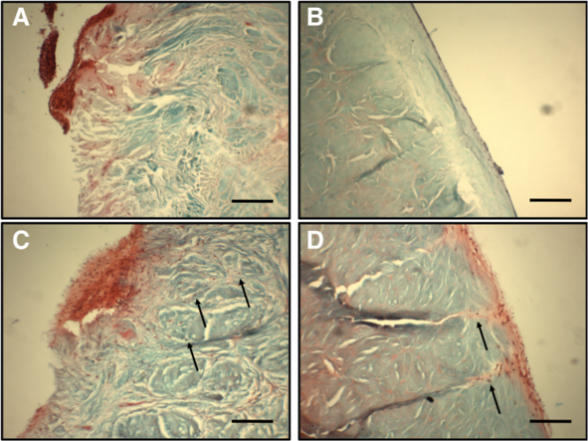
Safranin-O staining of reseeded meniscal samples. hASC seeded on a non-needle-punched decellularized meniscus within the **a** exterior “red-red zone” and **b** interior “white-white zone” of the meniscus. hASC seeded on a needle-punched decellularized meniscus on the **c** exterior “red-red zone” and **d** interior “white-white zone” of the meniscus. Collagen is stained in *blue*, and nuclei (*arrows*) are stained in *dark brown*. Greater cellular infiltration was observed in needle-punched samples. All scale bars = 200 μm

Histological analyses of the hASC-seeded meniscal allografts indicated that cells were able to proliferate on the periphery of the menisci, but decellularization alone did not allow for cells to migrate further than the surface of the menisci (Fig. 5a, b). With needle punching, cells were able to migrate through the pores deeper into meniscal tissue (Fig. 5c, d).

### Biochemical analysis

**Fig. 6:**
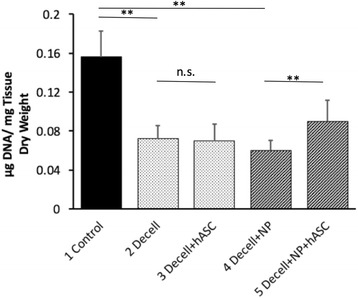
Subsurface DNA content of menisci normalized to dry weight after 4 weeks of culture. DNA content of menisci was significantly reduced in decellularized menisci (**p < 0.01 decell vs. control and decell + NP vs. control). Recellularization did not increase DNA content in menisci that had not been needle-punched (n.s. *p* > 0.05 decell vs. decell + hASC). However, needle-punched menisci had increased DNA content when reseeded (**p < 0.01 decell + NP vs. decell + NP + hASC)

Biochemical quantification of DNA content between treatment groups (Fig. 6) showed a 54 % reduction in DNA in the non-needle-punched decellularized menisci when compared to the control (0.156 ± 0.0265 vs. 0.0719 ± 0.0139 μg DNA/μg tissue, *p* = 0.001). When the menisci were needle punched, there was a 61.5 % reduction in DNA content (0.156 ± 0.0265 vs. 0.0602 ± 0.0101 μg DNA/μg tissue, *p* = 0.0005). When menisci were not needle punched, reseeding with hASC did not increase DNA content within the menisci (0.0719 ± .0139 vs. 0.0699 ± 0.0176 μg DNA/μg tissue, *p* = 0.6853). However, when menisci were needle punched, reseeding with hASC significantly increased DNA content within the menisci (0.0602 ± 0.0101 vs. 0.0901 ± 0.0217 μg DNA/μg tissue, *p* = 0.0009).

### Material properties

Anterior and posterior values of the equilibrium moduli (10 % strain, 20 % strain) and dynamic moduli (10 % strain at 0.1/1/10 Hz, 20 % strain at 0.1/1/10 Hz) were not significantly different (per paired *t* tests), and the mean values are reported here for simplicity, although both values were used in the mixed-model analysis.

**Fig. 7:**
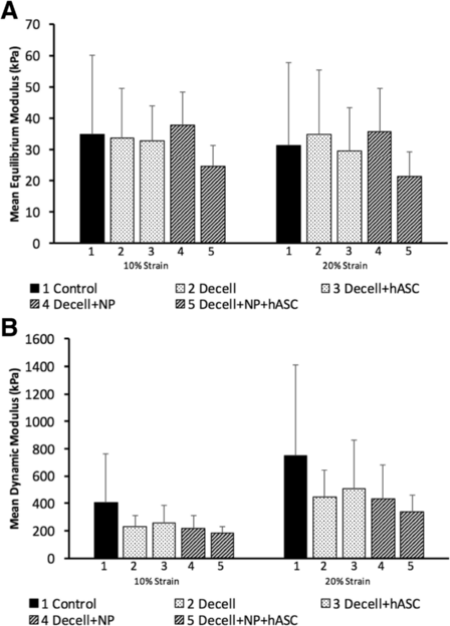
Decellularization, needle punching, and reseeding with hASC had no significant effect on meniscal material properties, assessed with **a** equilibrium modulus (mean over anterior and posterior samples) and **b** dynamic modulus (mean over all frequencies for anterior and posterior samples)

Decellularization did not significantly affect the equilibrium modulus, as the values in the four treatment groups were similar to those in the control group at both 10 % strain (32.2 ± 12.0 vs. 34.9 ± 25.3 kPa, *p* = 0.71) and 20 % strain (30.3 ± 15.1 vs. 31.4 ± 26.3 kPa, *p* = 0.88, Fig. 7a). Dynamic modulus tended to be lower for menisci in the treatment groups than in the control group for both 10 % strain (mean 219.4 ± 94.0 vs. 408.4 ± 352.9 kPa) and 20 % strain (mean 426.8 ± 245.1 vs. 748.6 ± 658.4 kPa), but these differences were not statistically significant at 0.1 Hz (10 % strain, *p* = 0.059; 20 % strain: *p* = 0.11), 1 Hz (*p* = 0.069, *p* = 0.12), 10 Hz (*p* = 0.092, *p* = 0.13), or for all frequencies combined (*p* = 0.066, *p* = 0.11, Fig. 7b).

Neither needle punching nor reseeding with hASC significantly altered either the equilibrium or dynamic moduli beyond the effects of decellularization. For needle-punched menisci only, reseeding with hASC tended to reduce the equilibrium modulus at 10 % strain, although this effect was not significant (*p* = 0.088).

## Discussion

Ideally, a meniscal allograft would be populated with a patient’s own autologous cells that could respond to mechanical stimuli and maintain the meniscus throughout the patient’s life. However, current meniscal allografts have high-density collagen networks that are difficult for cells to penetrate in vivo. In the current study, we have investigated the potential to improve human meniscal allografts by introducing additional porosity to the decellularized human menisci and seeding with hASC. We hypothesized that increased porosity would allow for increased autologous cellular infiltration into the scaffold.

Following a previously studied method of meniscal decellularization [23], we first verified that the menisci were decellularized and supported hASC cell growth. The meniscal allografts maintained their structure throughout the decellularization process, confirmed both on a macroscopic scale via gross observation and at the extracellular matrix level via SEM. In accordance with previous studies, decellularized menisci lacked defined nuclear staining, demonstrating the absence of cellularity. Immunohistochemistry of fresh, cryopreserved, and frozen human menisci have shown that the presence of human leukocyte antigen and A, B, and H blood group antigens can complicate a meniscus allograft transplant [21, 38]. In this study, we have successfully removed donor cells while maintaining the overall structure of a human meniscal allograft. Reseeding the decellularized allograft with hASC showed promising results, as cells remained viable and even organized parallel to the structure of the collagen fiber extracellular matrix within the meniscal allograft. The viability of the hASC was maintained on both intact and needle-punched menisci. The ability of hASC to align parallel to the native collagen fibers in the meniscal allograft is promising in that it may allow for future collagen production by hASC within a meniscal allograft. Cell orientation has been previously shown to determine alignment of a cell-produced collagen matrix [39, 40].

Histological evidence showed an increase in cellular infiltration in needle-punched menisci compared with non-needle-punched menisci. The increased porosity introduced channels through which the hASC can migrate into the interior of the scaffold, which is critical for the living scaffold to integrate fully and maintain tissue homeostasis. hASC seeded on scaffolds that lacked needle punching clustered on the surface of the meniscus, but did not penetrate into the interior of the scaffold. Altogether, this demonstrates that the introduction of additional porosity is necessary for allogeneic meniscal transplants to permit the infiltration of autologous cells.

Biochemical analysis of DNA content further elucidated the extent of decellularization and recellularization in the allografts. DNA content was significantly decreased (*p* < 0.01) in both menisci with no needle punching and needle-punched menisci. Although the menisci still contained some nuclear material, we obtained 54 % reduction in DNA content, which was highly consistent with the 55 % reduction that had previously been reported using this decellularization method in ovine menisci [23]. It has been postulated that nuclear material remains in the menisci through hydrostatic interactions with the tissue, even when cellular content is absent [23]. A slightly lower DNA content was observed in the needle-punched menisci with a 61 % reduction. Since needle punching took place after the decellularization process, this suggests that the needle punching allowed residual DNA fragments to wash out during the 4-week incubation period. During the recellularization process, no increase in DNA content was observed in the non-needle-punched samples. However, the needle-punched menisci did significantly increase (*p* < 0.01) in DNA content when reseeded. Since the biochemical samples were taken from the interior of the meniscus, this suggests that cells were unable to migrate into the non-needle-punched menisci but were able to penetrate into the needle-punched menisci. Although DNA content did not reach levels of native tissue in the reseeded needle-punched menisci, future optimization of this method could lead to more efficient infiltration of hASC. Herein, we demonstrated that the seeded hASC can migrate into the interior of the needle-punched allograft. Cellular content in the central zones may increase with longer culture or after in vivo implantation, which will be the subject of future investigations.

Both static and dynamic compressive material properties were not altered significantly by treatments used to prepare the meniscal allografts. Relative to control samples, menisci that were decellularized, needle punched, and/or reseeded with hASC had similar equilibrium and dynamic moduli in unconfined compression, regardless of the anatomical location (anterior, posterior), strain level (10 %, 20 %), or dynamic testing frequency (0.1, 1, 10 Hz). A previous study in fresh human meniscus showed that dynamic compressive modulus was positively correlated with glycosaminoglycan content and negatively correlated with water content. Therefore, the trend for decreased modulus with decellularization may indicate a slight loss in collagen content, increase in water content, or both within the allografts [41]. The moduli values in this study were similar to those reported in other studies of axial unconfined compression in human meniscus [41, 42]. While some studies have measured differences in moduli for different locations within the meniscus (anterior, central, posterior), others have measured similar properties in anterior vs. posterior samples [42], as shown here. Our material property testing results are consistent with a recent study in which it was shown that laser drilled menisci exhibited a slight decrease in Young’s modulus and instantaneous stress but remained within a physiological range [43].

A limitation of the current study is that only one needle-punching configuration (1-mm^2^ spacing with a 28 G microneedle) is tested. Future studies should optimize spacing and needle diameter to allow cellular infiltration while minimizing disruption of the natural meniscal architecture. In addition, future in vivo studies must be carried out to assess the potential for clinical translation. We expect that the hASC introduced into the scaffolds would allow for enhanced remodeling of the meniscus once the scaffold is transplanted in vivo.

## Conclusions

In this study, we observed the increase in hASC infiltration into human meniscal allograft, without significant alterations in the compositional or mechanical integrity of the scaffold. Since hASC can be easily isolated in large quantities for autologous use, the hASC could be an ideal cell source to repopulate an allograft scaffold before transplantation into the patient. Successful tissue engineering utilizing hASC would allow meniscal allograft transplantation to be transformed by improving and extending the life of the transplant, leading to a large clinical advancement in the treatment of patients with meniscal deficiency.

## Abbreviations

CGM: Complete growth medium
DAPI: 4,6-Diamidino-2-phenylindole
hASC: Human adipose-derived stem cells
MSC: Mesenchymal stem cells
NP: Needle-punched
SEM: Scanning electron microscopy
